# Loxoprofen‐induced bullous fixed drug eruption

**DOI:** 10.1002/jgf2.288

**Published:** 2019-12-06

**Authors:** Takayuki Yamada, Hideta Sakemi

**Affiliations:** ^1^ Asunaro Clinic Takasaki City Japan; ^2^ Rakuwakai Otowa Hospital Kyoto Japan

## Abstract

A 49‐year‐old housewife with a long‐standing migraine presented with "spells" of intensely itchy, well‐circumscribed, erythematous patches over the flexor aspect of her left wrist and palm repeatedly for the last 15 years. Detailed history revealed her oral loxoprofen use for migraine headaches preceding rash development. Although a patch test was negative, inadvertent ingestion of the drug by the patient reproduced the rash within a few hours, thereby establishing the diagnosis of loxoprofen‐induced bullous fixed drug eruption.

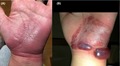

1

A 49‐year‐old housewife with a long‐standing migraine presented with "spells" of well‐circumscribed, erythematous patches over the flexor aspect of her left wrist and palm (Figure 1A). She repeatedly had similar rashes, once with hemorrhagic bullae (Figure 1B), which always started abruptly with an pinprick sensation followed by an intense itch and erythema at exactly the same spot, up to three times a year for the last 15 years. With or without treatment, the itch subsided in 1‐3 days, and the erythema turned dusky, then desquamated, and finally disappeared in 2‐4 weeks. Detailed history revealed her use of loxoprofen 60 mg orally for migraine headache up to 2 days prior to the development of the rash, which she had never thought rash‐related. A patch test was performed in the patient using loxoprofen tape (Loxonin® Tape; Daiichi Sankyo Ltd) 6 weeks after resolution of an acute flare. The tape was applied over her left wrist, covering both the involved and uninvolved areas simultaneously for 48 hours. After the tape was removed, skin change was observed on day 2, day 3, and day 7. Although the patch test could not induce the skin lesion, an inadvertent ingestion of the drug (Loxonin® Tablet; Daiichi Sankyo Ltd) by the patient resulted in rash development within a few hours, thereby establishing the diagnosis of loxoprofen‐induced fixed drug eruption (FDE).

**Figure 1:**
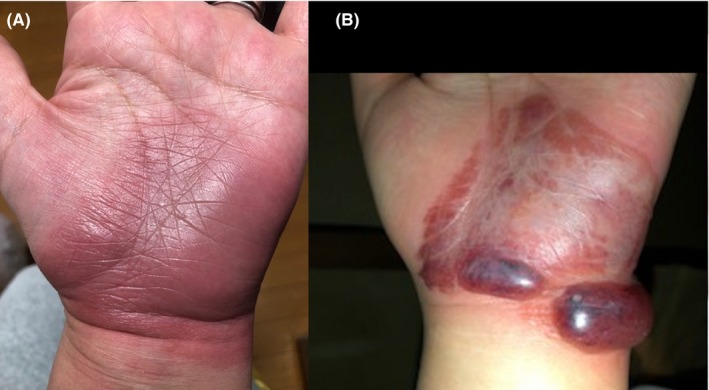
A, The patient's left wrist and palm at presentation. B, A photograph of the skin lesion taken by the patient several years previously

Fixed drug eruptions are allergic hypersensitivity responses to a drug exposure and are characterized by localized well‐circumscribed lesions that reappear in the same location upon re‐exposure to the same drug or allergen. CD8+ T cells with an effector‐memory phenotype are fixated at the lesion for years and remain quiescent until stimulated by the causative agents.^1^ A patch test may not deliver sufficient amount of the agent to the T cells, or the agent may need to have been metabolized to a form that can stimulate the T cells, thereby making oral challenge test more sensitive than patch testing, as was in this case.

The typical presentation of FDE is distinct. In the acute phase, FDEs present as usually solitary, well‐demarcated, circular, edematous, violaceous hyperpigmented macules or patches. Pruritus and a stinging sensation are common, while systemic symptoms, such as fever and malaise, are typically absent. Vesiculation and blistering are common, but intensely hemorrhagic bulla formation is rarely reported. Hyperpigmentation persists long after the acute phase subsides.

Patients often fail to associate drug ingestion with FDE development because of a relatively long—on average, two days—latency period.^2^ Therefore, detailed drug history in addition to recognition of the characteristic rash is the key for diagnosing FDEs.

## CONFLICT OF INTERESTS

The authors have stated explicitly that there are no conflicts of interest in connection with this article.
